# Barriers and facilitators for implementation of a digital referral algorithm for inflammatory arthritis - a qualitative assessment in patients and caregivers

**DOI:** 10.1186/s12875-022-01858-w

**Published:** 2022-09-26

**Authors:** Elke van Delft, Ruben Bos, Patricia Pennings, Mieke Hazes, Deirisa Lopes Barreto, Angelique Weel-Koenders

**Affiliations:** 1 Department of Rheumatology, Maasstad Hospital, Maasstadweg 21, 3079 DZ Rotterdam, The Netherlands; 2 National Association ReumaZorg Nederland, Nijmegen, The Netherlands; 3 Department of Rheumatology, Erasmus Medical Center, Rotterdam, The Netherlands; 4 Health Technology Assessment, Erasmus University, Rotterdam, The Netherlands

**Keywords:** Qualitative research, Implementation, Integration, Patients’ perspective, Caregivers’ perspective

## Abstract

**Purpose:**

Difficulty to recognize inflammatory rheumatic diseases (IRD) in a primary care setting leads to late referral to secondary care. An evidence-based digital referral algorithm can support early referral, yet implementation in daily practice only succeeds with support of end users. We aim to understand the context of implementing a digital referral algorithm and explore the potential barriers and facilitators to implementation.

**Methods:**

This qualitative study comprised focus groups and an online survey. Focus groups were performed with patients from outpatient rheumatology clinics. Surveys were sent out to general practitioners and rheumatologists distributed over The Netherlands. The presented digital referral algorithm originates from the JOINT referral study. Thematic analysis was used with inductive and deductive approaches.

**Results:**

In total 26 patients participated distributed over three focus groups, and 215 caregivers (104 rheumatologists, 111 general practitioners) filled out the survey. Both patients and caregivers endorse the need for early referral, and recognize the perceived benefit of the digital algorithm. Potential barriers include the complexity of currently included questions, and the outcome lacking information on what to do with no risk of IRD. In order for implementation to be successful, the inclusivity, accessibility, content and outcome of the algorithm are considered important themes.

**Conclusion:**

Successful implementation of a digital referral algorithm needs a systematic multi-facetted approach, considering the barriers and facilitators for implementation as discussed. Since the majority of identified barriers and facilitators was overlapping between all stakeholders, findings from this study can reliably inform further decision strategies for successful implementation.

**Supplementary Information:**

The online version contains supplementary material available at 10.1186/s12875-022-01858-w.

## Introduction

In The Netherlands general practitioners (GPs) have a crucial role as gatekeepers. Despite the specific complaint a patient has, GPs decide whether referral towards secondary (or more specialized tertiary) care is considered necessary or if the ailment can be controlled within primary care. Currently there is a dilemma present within the referral strategy for inflammatory rheumatic diseases (IRD). Since IRD is difficult to recognize by GPs, a lengthy referral delay leads to late diagnosis and ultimately decreased quality of life on the one hand [1, 2]. On the other hand, promoting early referral leads to a high percentage of inadequate referrals [3, 4].

One of the most potent ways to increase appropriateness of referrals within the increasing number of patients with musculoskeletal complaints [5] is a structured referral sheet (e.g. algorithm) [6]. By using structured referral sheets, the appropriateness of referral improves significantly, alongside it showing good potential to improve cost-effectiveness. For IRD several validated referral sheets have been developed, like the CaFaSpA for axial spondyloarthritis [7], the PEST for psoriatic arthritis [8] and the CARE for early arthritis [9]. Recently, we have validated that the CARE is suitable to be used for all forms of IRD [10], and we also published a composite algorithm for all forms of IRD [11]. The actual implementation of such a (digital) healthcare innovation is however a complex process [12] and needs preceding research. The main constraint to an optimal implementation process is lack of patient and provider involvement [13–19]. To our knowledge, both patient and provider involvement are lacking so far when considering implementation of a digital referral algorithm to identify IRD patients. For successful implementation, it is essential to identify the barriers and facilitators that might affect the adaptability and implementation [20, 21], however these are considered not to be influenceable. Therefore it is also important to identify factors that can be actively addressed to facilitate implementation, e.g. implementation factors. Feedback and clarity from patients and healthcare providers on awareness, support base and usability are expected to facilitate the development and implementation of a valid strategy [22].

Qualitative research has been proven an effective methodology to draw upon attitudes, experiences and reactions from end users for successful implementation [23]. A constructivist grounded theory approach to qualitative research is proficient to understand both patients’ and caregivers’ perspectives on our referral strategy. Focus groups are considered a well suited method for the purpose of understanding the perspective of patients with musculoskeletal complaints because of the dynamic interaction within the group leading to fuller and deeper discussion and triggering of new ideas [24, 25]. A survey is considered useful and feasible to understand the caregivers’ perspective, since they are often hard to engage due to their scarce time [26].

Therefore, in this qualitative study, we aimed to explore whether there is a support base for a digital IRD referral algorithm among patients, general practitioners and rheumatologists. Subsequently we investigated the barriers and facilitators from a patient and a caregiver perspective regarding awareness, usability, accessibility and acceptability of the referral algorithm.

## Methods

### Study design

Qualitative research using a constructivist grounded theory approach was chosen as we sought to understand patients’ and caregivers’ perspectives on our referral algorithm [27]. We were interested to investigate the support base and identify the barriers and facilitators that might affect the implementation. This qualitative study consisted of both focus groups with patients [24, 25] and online surveys among caregivers [26], and was conducted between December 2020 and June 2021. This study was approved and considered not to be subject to the full extent of the Medical Research Involving Human Subjects Act by the Medical research Ethics Committees United (MEC-U) situated in Utrecht, the Netherlands (registration number W.20.260).

### Sampling and recruitment

Patients were selected on inclusion criteria of being 18 years or older, having visited the rheumatologist for suspicion of having an IRD, and being able to understand and communicate in Dutch. For the first focus group, we collaborated with the Dutch patient organization for people with rheumatic diseases (National Association ReumaZorg Nederland) to select patients with an IRD diagnosis. For the second and third focus group we sampled patients who had been referred in 2020 to the outpatient rheumatology department of the Maasstad hospital. Patients were invited to participate via phone and included in the focus group after informed consent.

Participants were sampled through purposive sampling. Based on the iterative analysis of the data from the focus groups, we allowed for purposive and theoretical sampling of patients within each subsequent focus group. Moving back and forth between sampling, data collection and data analysis, allowed us to select participants who could best contribute to the developing theory and explore certain co-constructed themes in greater depth with each subsequent focus group [28]. Participation in the study was voluntary and no compensation was given.

Caregivers were selected on inclusion criterion of being currently employed as a GP or rheumatologist in The Netherlands, therefore being able to understand and communicate in Dutch. Professional networks were addressed to recruit potential participants. For the rheumatologists, the online survey as set up in Qualtrics was distributed by the Dutch Society for Rheumatology (NVR). For the GPs the survey was distributed by KOEL foundation, an organisation focused on connecting and enhancing primary care in the region of South-Holland, and by a subdivision of the Dutch GP society (NHG) called NHGDoc. The surveys were sent by email, a reminder email was sent after 2 weeks, and after 4 weeks the survey was closed.

### Digital referral algorithm

The digital referral algorithm used for this study [11] was based on a combination of recently developed and validated referral questionnaires for axial spondyloarthritis (CaFaSpA [7]), psoriatic arthritis (PEST [8]) and early arthritis (CARE [9]). The composite algorithm consists of five to eight questions, specific to the main complaints of a patient (Supplementary file 1B). After completing the algorithm, which will take approximately 2 min, a total score will be calculated and compared to a threshold value. When a patient is considered to be at risk for IA, an advice is given for referral towards a rheumatologist. Currently the algorithm is not yet in active use, however ideally it will be made available online for patients to use by themselves.

### Data collection

#### Focus groups

Focus groups were conducted in a live or online setting with a group size of 6-10 patients. The first one was conducted in a live setting in Utrecht, central of The Netherlands. Following focus groups were hosted via the online communication platform Microsoft Teams due to Covid-19 regulations. In the week prior to the focus groups patients were asked to provide information on participant characteristics such as age, gender, education, occupation in daily life, duration of complaints, and diagnosis to allow for theoretical sampling in following focus groups.

Focus groups with patients were guided by two researchers, of whom one (ED) had the role of moderator and focused mainly on asking questions and prompting the group discussion. The other had the role of observer (MD), and focused on observing the behaviour of patients, keeping track of time, sticking to topic, taking notes and recording the focus group. Both moderators were female PhD students in the field of rheumatology at the time of the study, and were qualified in qualitative research. Moderators were independent from the patients’ care cycle and priory unknown to the patients.

All focus groups started informal during which patients and moderators introduced themselves. After this introduction, the purpose of the study was discussed. Patients were assured of the confidentiality of the discussions and that participation was voluntary with the option to withdraw at any time. Furthermore, their permission to audio-record the session was confirmed and patients were assured of anonymous verbatim transcription. Patients were provided with a screenshot of the algorithm as presented in Supplementary file 1B as well as a demonstration of use of the algorithm by the researcher. The semi-structured question guide consisted of 12 main questions to explore patients’ opinions and experiences with suffering from joint complaints and the choice to visit a GP from their own patient journey. Questions were also targeted on how patients wanted to be supported within their journey and how our digital algorithm might be helpful to overcome any obstacles (Supplementary file 1A). In total one-and-a-half to a maximum of 2 h was set for each focus group discussion. Transcripts from the focus groups were returned to patients to allow them to provide feedback, comments or corrections.

#### Surveys

To gain insight into the caregivers’ perspective on our digital referral strategy for patients to use at home we used an online survey. The survey was based on the Measuring Instrument for Determinants of Innovations [20] and set up in Qualtrics. A copy of the proposed algorithm was provided [9]. Questions were asked including 5-point Likert scale questions, with a score of one indicating total disagreement and five indicating total agreement with proposed statements, and open-ended questions with a free-text format to obtain more in-depth qualitative data [29] (Supplementary file 1C).

### Data validation and analysis

For demographic variables the number and percentage will be presented on categorical variables. The mean and standard deviation (sd) will be presented for continuous variables, or median and range for continuous variables with a small number of observations or non-normally distributed data. Education of patients was subdivided into low (none or elementary school), intermediate (secondary or intermediate vocational school) and high (higher vocational education or university).

**Fig. 1:**
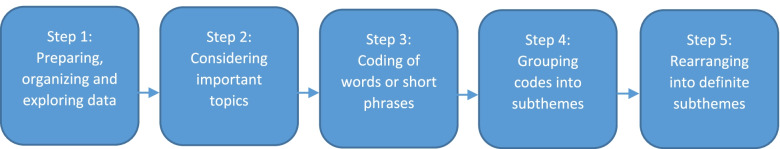
Five steps of thematic analysis

All qualitative data was analysed by two independent researchers (ED,RB) following a thematic analysis approach consisting of five steps (Fig. 1) [30]. The emergent recurring and/or salient themes formed the basis for recommendations for further development and implementation of the digital referral algorithm. Facilitators, barriers and implementation factors were chosen as main themes in advance. Two researchers (ED,RB) individually read the transcripts several times to become familiar with the data and develop a sense for emergent topics (step 1) [31]. Topics were considered to be important when they either recurred frequently in the transcript, or when participants took extreme positions (step 2). Words or short phrases that symbolically assigned a summative, salient, essence-capturing or evocative attribute for a portion of the data were manually coded (step 3) [32]. After coding the entire transcript, the codes were grouped into subthemes of topics that overlapped or supplemented each other (step 4). This followed an iterative process using a constant comparative method, allowing for both an inductive approach, where new subthemes were created based on new information, and a deductive approach, where new information was divided into existing subthemes (step 5) [33]. The two researchers critically appraised whether new data yielded new or redundant information. When no new analytical information arose anymore and the study provided maximum information on the specific end user perspective, no new participants were included anymore [34].

The emerged subthemes were elaborately discussed between the two researchers and agreement of ascription of codes was assessed by the kappa statistic. A number of direct quotes from participants supporting the subthemes are included in the results. Following the quote, there is an indication of which participant stated it (P = patient, F = focus group, R = rheumatologist, G = general practitioner). Remaining 5-point Likert scale questions were analysed and presented in a descriptive manner.

The qualitative analysis software MAXQDA Analytics Pro 2020 (Release 20.3.0) was used to organize and support the coding process. The consolidated criteria for reporting qualitative research (COREQ) guidelines will be used to describe the findings [35].

## Results

In total three focus groups were conducted until data saturation was achieved. The focus groups consisted of ten, six and ten patients respectively. The focus group discussions had a mean duration of 102 minutes (range from 85 minutes to 115 minutes).

Mean age (range) of patients was 52 (25–73) years and 20 (69%) were female (Table 1). As a result of the theoretical sampling, all patients in the first focus group were diagnosed with an IRD, 83% in the second and 40% of patients in the third focus group. The most common IRD diagnosis was rheumatoid arthritis, followed by axial spondyloarthritis and psoriatic arthritis. The most common non-IRD diagnosis was fibromyalgia.

**Table 1:** Demographic characteristics of patients

	Focus group 1 (*n* = 10)	Focus group 2 (*n* = 6)	Focus group 3 (*n* = 10)
Age in years, median (range)	63.0 (25–73)	45.0 (25–68)	51.5 (26–73)
Female, n (%)	6 (60)	5 (83)	7 (70)
Education, n (%)			
Low	1 (10)	1 (17)	1 (10)
Intermediate	2 (20)	2 (33)	6 (60)
High	7 (70)	3 (50)	3 (30)
Paid work, n (%)	2 (20)	4 (67)	5 (50)
IRD, n (%)	10 (100)	5 (83)	4 (40)

### Patients’ perspective

#### Current process of regular care referral

In some cases, referral to a rheumatologist was scheduled directly after the first GP visit, mostly related to increasing patient age (> 50 years) and a family history of IRD. However, the majority of patients experienced a diagnostic delay that was mostly attributable to a GP’s delay, caused by a lack of knowledge on IRD, especially in young MSC patients and as cause of autoimmune diseases*. “That delay in referral caused me nine years of trouble. Joint damage, barely being able to work, to sleep, experiencing a lot of stress, anxiety attacks; a lot of trouble.” (P7F1)* Reasons for patient delay were the following: complaints considered not severe enough, thought to bother their GP, and a fear to be labeled with a lifelong diagnosis. Patients never hesitant to consult their GP were mostly elderly (> 55 years).

Patients often had sought for information online prior to consulting their GP. *“Many people google their symptoms. You want to have an explanation, something that can explain your pain.” (P6F2)* However, patients did not find the right information and considered the currently available information to be too broad and extensive.

#### Digital referral algorithm

**Fig. 2:**
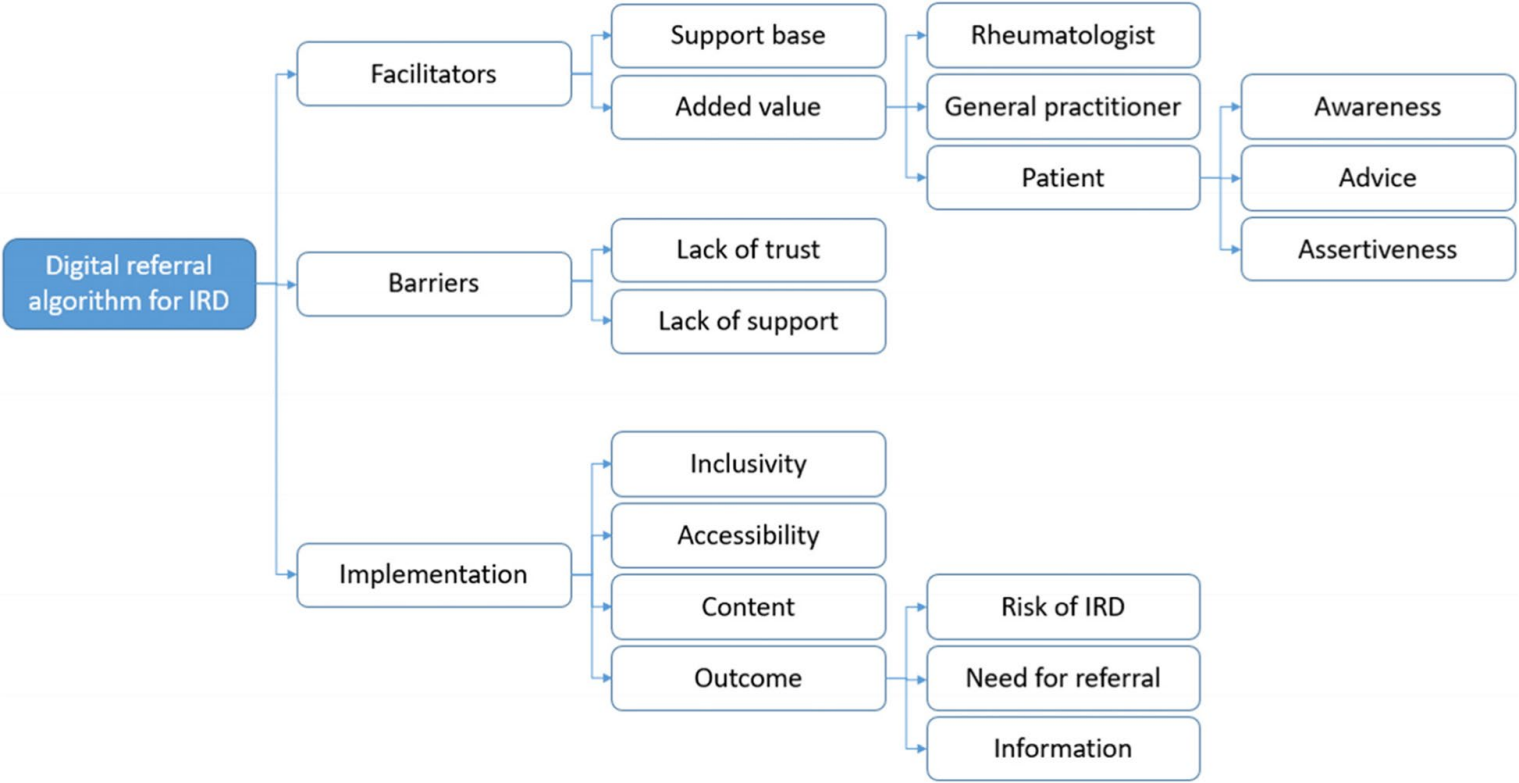
Overview of the emerged (sub)themes as used for analysis

Within the main themes of facilitators, barriers and implementation factors for our proposed digital referral algorithm, we deducted subthemes from the focus groups (Fig. 2). Kappa statistic for identifying the subthemes was 0.90, indicating almost perfect agreement between the two researchers. The need of a referral algorithm was felt consistently within all groups, although within each focus group different facilitators and barriers were reported.

#### Facilitators

Overall patients stressed the need for a supportive tool like our digital referral algorithm, in the light of organizing care centered around the patients*; “In this way it is the patient who is central in his or her own care cycle, which is consistent with the time we live in right now.” (P3F1)* This tool might empower patients to speak up for themselves, increase their credibility, and lead to patients becoming allies with their GP. *“This tool allows us to support our own general practitioner to come to a diagnosis.” (P4F2).*

The added value for patients in the use of this referral algorithm was seen in enhancing awareness and knowledge of IRDs, accompanying symptoms and related specialist. A positive referral advice was considered a wake-up call to patients and an encouragement to seek help. A negative referral advice is also considered helpful; *“If the tool were to turn out negative, I would start searching in other directions.” (P2F3)* Patients stated the referral algorithm might also increase GPs’ knowledge about IRD, related symptoms, and the possible relation with other autoimmune conditions. *“How amazing would it be if your general practitioner, based on this tool, could skip some steps and initiate the right next steps immediately.” (P3F3).*

#### Barriers

The barrier most often indicated was the questions used in the algorithm being too complicated and possibly leading to misunderstanding and answering erroneously. Depending on the outcome of the tool, certain diagnoses might be ruled out too easily. As an additional barrier, patients think that not every GP is open to or accepts information that a patient has found online, but instead merely relies on his or her own knowledge, based on training and experience.

Of all patients, one of them expressed to see no use in this referral algorithm at all. *“In all honesty I must say that I do not see it with this tool. I think when people have to fill it out themselves, that would not work. There is too much information, I think people would only get more confused.” (P9F3)* The patient that stated these doubts, also expressed not to use google anyway and was one of our oldest included patients.

#### Implementation

All but one patient stressed that this referral algorithm might be of great help and should therefore be very inclusive by assuring easy comprehensibility and easy accessibility for everyone. Patients also emphasized the idea to implement this algorithm for use by the general practitioner, instead of use by patients themselves. Broadening of the outcome of the algorithm, by adding additional advice on recommended next steps especially with a negative referral advice, was considered necessary by patients to fulfil successful implementation.

### Caregivers’ perspective

The survey was filled out by 104 rheumatologists (range 30–63 years, 52% female) and 111 general practitioners (range 26–74 years, 46% female) (Table 2). Since results on the usefulness of the referral algorithm were largely consistent between both groups of caregivers, results are bundled.

**Table 2:** Demographic characteristics of caregivers

Survey	Rheumatologists (***n*** = 104)	General practitioners (***n*** = 111)
Age in years, mean (sd)	44.9 (8.9)	43.3 (12.0)
Female, n (%)	52 (74)	46 (61)
Working at academic hospital, n (%)	10 (13.9)	N/A

*N/A* Not applicable

#### Current process of regular care referral

Rheumatologists estimated that 31% of all referred patients is diagnosed with an IRD. GPs expected that an IRD is diagnosed in 40% of referred patients. Almost all caregivers mentioned that the high frequency of non-IRD compared to IRD is at least partly due to a lack of knowledge on (symptoms of) IRD among patients. Caregivers underlined that patients look for information on their complaints online prior to consultation. However, there is little trust among caregivers in the currently available information platforms for patients. Overall, both rheumatologists and GPs indicated the need for improvement of appropriate referral.

#### Facilitators

Both rheumatologists and GPs indicating to see added value, often saw this for multiple stakeholders, however mainly for patients. Caregivers stated that the referral algorithm may create awareness and increase knowledge on IRD and accompanying symptoms among patients. It may help patients to order their thoughts, be well-prepared before a medical consult, and create a targeted question for their GP visit. Caregivers expect that use of the algorithm might temper patients’ expectations; *“Expectations may become more clear, most importantly on what is NOT necessary.” (G10).*

Caregivers suspect that use of this algorithm may lead to a decreased number of GP consultations and a reduction of inappropriate referrals to the rheumatologist. The algorithm might support GPs in the decision-making process for referral, supporting early referral and start of treatment in patients with high risk for IRD. Next to that, the algorithm might empower the GP’s decision of not referring a patient by convincing the patient when referral is unnecessary. *“For general practitioners it will become easier to assure a patient that referral is not necessary when the tool is indicative of a negative referral advice.” (R88)* Some of the GPs specifically mentioned that the use of this algorithm can stimulate shared decision making between patient and GP.

#### Barriers

Of the rheumatologists, a third declared to not support the idea on implementing a referral algorithm, since firstly non-IRD patients should visit outpatient rheumatology care because the rheumatologist is the preferred medical specialist for any (persistent) joint complaint with a non-traumatic cause. Secondly, patients are better off with a face-to-face caregivers’ consultation. *“Patients want to see a doctor because of their complaints, whether them being inflammatory or not.” (R54)* Although the vast majority of GPs did support the idea on implementing a digital referral algorithm, few were concerned whether patients will follow the advice from a digital algorithm*. “A part of referrals is due to the fact that patients get a certain idea into their heads and they can only be reassured once seen in secondary care.” (G28).*

The fact that the referral algorithm is ought to be used by patients themselves created a lack of trust, because it is too complicated for patients to fill out, especially questions regarding physical complaints, like swelling, which is hardly recognized correctly by patients. Caregivers, particularly GPs, state; *“The biggest pitfall is that patients fill out the desired answers needed in order to receive a positive referral advice.” (G18)*. Caregivers wish elaborate testing and validating of the algorithm to address sensitivity and specificity.

#### Implementation

Caregivers indicated that the algorithm should be inclusive for everyone, available also offline and in languages other than Dutch with special attention on low health literacy. Caregivers expressed major concerns about whether the algorithm is suitable to implement for patients to use at home. The solution considered best suitable was to implement this algorithm for use within primary care, since the algorithm could support GPs in the decision-making on the need for referral, next to enhancing shared decision making. It should then be made available within already existing guidelines or systems.

*“The referral tool might be very convenient for use by a general practitioner. The way it is now, that would work.” (R43)* However, a small portion of rheumatologists as well as GPs indicated that they would like to add a question on the presence of red or warm joints, as well as on joint complaints being symmetrical. As essential component of the outcome, more information for patients on advised next steps in the care cycle was suggested; *“The tool should provide an answer to the question: ‘Where do I find the help that I need?’.” (R81)*). Information can be delivered through links to already existing, reliable online information.

## Discussion

In this qualitative study we established that there is indeed a need for a digital referral algorithm indicating the risk of IRD and need for referral. This is supported by patients, GPs and rheumatologists, perceiving it as a helpful innovation in order to increase the appropriateness of referrals to the rheumatologist. The fact that the added value is seen by and for all included stakeholders indicates that a support base is present which can be a facilitator in the adoption phase of this healthcare innovation. In fact, perceived benefit of an innovation by the stakeholders involved is considered to be the most powerful facilitator [35]. On top of that, from our results it was shown that all stakeholders felt a need for change resonating with the proposed strategy, which is also a known important facilitator [36].

Both patients and caregivers labeled the narrowness of the outcome of the proposed algorithm as a major barrier. Important information for the patients and GPs is missing in the current outcome, especially when the outcome of the algorithm is that there is no risk of an IRD. To increase usability, the algorithm should be able to advice on possible treatment options in primary care when providing a negative referral advice. For many non-inflammatory joint complaints, elaborate guidelines for management within primary care already exist that can be incorporated into the outcome of our referral algorithm [37, 38].

A remarkable finding is the discrepancy between expectations of patients and rheumatologists at some points. For example, participating rheumatologists claimed to know for sure that all patients with musculoskeletal complaints always want to have a face-to-face consultation with a rheumatologist, no matter the complaint. This was mentioned as one of the main barriers by the one third of rheumatologists who did not support the idea of implementation. On the contrary, patients state they just want to receive the right care at the right place, no matter what the right place is. On top of that, we have proven that quality of life significantly increases if non-IRD patients are being withheld from unnecessary outpatient care [39]. This highlights the importance of organising healthcare towards more personalised healthcare [40]. Patients within the present study are ready for this change, and very willing to take more control over their own patient journey. Next to patients, also GPs acknowledge the added value of shared decision making. Shared decision making is increasingly advocated as an ideal model of decision making during the medical encounter, as it has shown to improve patients outcomes and increase benefits for both clinicians and the healthcare system [41, 42]. Therefore, to convince the hesitant one third of rheumatologists, personalised healthcare and shared decision making should be given a more prominent role within healthcare policies.

Our initial idea was to implement this algorithm for patients to use at home. The qualitative analysis indicated that this approach should be reconsidered. GPs themselves admit to have doubts on the necessity of referral in about a quarter of all patients with musculoskeletal complaints. Therefore use of this referral algorithm by GPs might be convenient in supporting GPs in making a decision on referral.

Previously, referral tools for use by patients have been considered effective in increasing appropriateness of referrals, for example in patients with axial spondyloarthritis, one of the most common forms of IRD [43]. However, it has been noted that referral tools for use by patients should be applied supplemental to a referral tool for use by the GP. The referral algorithm for use by the GP should typically be implemented first [43]. In order to do so, it is essential that the algorithm is incorporated into GP information systems already in use in daily primary care practice to increase awareness and accessibility. Participating GPs often offer that it should be integrated into their GP information system.

The main strength of this study is the high number of responses gathered on the caregivers’ surveys. Since qualitative research is a method with high information richness of the data, mostly 75 responses to open ended surveys per group are sufficient to provide reliable results [44], where we collected over 100 responses per group. Especially noteworthy is the fact that almost 40% of all Dutch rheumatologists participated in our survey. Rheumatologists and GPs were selected completely at random, without any prior selection on age, seniority and affinity with digital innovations. Therefore, this gives a good reflection of the overall field of caregivers dealing with patients with musculoskeletal complaints, the population in which our algorithm is ought to be used.

Next to the caregivers’ perspective, we were also able to include a broad patient perspective. It is agreed upon that patient participation within healthcare is beginning to play a more important role [45]. A review of the literature reveals that participation of patients with musculoskeletal complaints in healthcare is associated with improved treatment outcomes and higher rating of the quality of care [45, 46]. Since patients were included with no prior selection on age, diagnosis and affinity with digital innovation, they are a good representation of the overall population of people with musculoskeletal complaints.

The qualitative methodology allows for several other strengths. The designed open-ended questions from both qualitative research methods were specific enough to yield coherent responses across respondents, yet broad enough to invite a spectrum of answers [47]. The combination of open and closed questions in the surveys allowed for efficient collection of valid data within closed questions, with the possibility of immediately reassuring that all relevant issues have been covered using the open ended questions [48]. The chosen questions within the survey were focussed on implementing our algorithm for patients to use at home, whereas in hindsight it may have been more useful to include questions on use of this algorithm by caregivers.

The iterative design of this study facilitated a more comprehensive insight in the perspectives of the stakeholders with regard to the proposed algorithm. Through verification of stakeholder claims with other stakeholders, validity and data saturation were enhanced. The rigorous thematic analysis approach has allowed to produce trustworthy and insightful findings [49]. Transparency of the results is ensured by providing direct quotes from participants.

What might be considered a limitation, is that two of the three focus group discussions had to take place online. Online focus groups might allow for less interaction and less perception of non-verbal communication [50, 51]. However, recently it is shown that online focus groups also have an interactive character and bear a strong resemblance to offline focus groups [52]. Since online focus groups are not bound by geographical location, it allows patients to participate from the comfort of their own location of preference, which may lower the threshold for patients with musculoskeletal complaints to participate. Online focus groups are well suited to gather a first reaction of respondents to a new healthcare innovation [53]. We believe that the weaknesses and benefits of online focus group discussions outweigh each other and the fact that no differences were found between the different focus group settings in the present study underlines that.

Another limitation might be that the results from our study are only transferable to settings with a similar healthcare system; in which primary care consultants act as gatekeepers to refer to secondary care. Since practice patterns for musculoskeletal and rheumatic diseases are distinct from other specialists [19], it is questionable whether the current results are directly transferable to other fields of healthcare. Lastly, the fact that researchers study a tool they have developed themselves may also be considered a limitation. However, during the focus groups one of the moderators present had not been involved during development of the algorithm, and is therefore considered to be independent.

Based on this study, we have some recommendations for clinical implication. Since the analysed digital algorithm is considered not suitable for patients to use at home, the focus shifts towards sufficiently supporting GPs in the decision-making on referral for patients with musculoskeletal complaints. Conveniently, the content of the algorithm as presented in this study is appropriate for use by primary care clinicians. The outcome of the algorithm however needs to be expanded with information on treatment options for non-inflammatory complaints within primary care. To do so we recommend to incorporate information already available, for example at the Dutch College of General Practitioners [38]. When these recommendations can be achieved, we encourage rheumatologists to place greater trust in the GP making the decision not to refer when sufficiently supported by the digital advice.

Another clinical implication is that IT facilities have to become supporting in realising sustainable, effective and patient centred healthcare at the right time and the right place. Until now, the multitude of systems that is available in primary care prevents working with a digital algorithm in real time. In order to modernize the systems towards patient-centred care, a sense of urgency must rise within primary care consultants to make changes happen [54]. Within this study, we have shown that GPs underline the need for change, and are very willing to work with such an innovation on the condition that it can be integrated within their current systems.

While the proposed algorithm and its included referral sheets have been validated [10], future research may be conducted focussing on validation of this referral algorithm in an independent, unselected primary care population, when in fact used by general practitioners. Within this validation, both sensitivity and specificity of this algorithm when used by GPs need to be established. Next to that, the cost-effectiveness of using the proposed referral algorithm should be investigated and compared to current usual care.

Concluding, patients, rheumatologists and general practitioners express the need for a distinctive referral algorithm indicating the risk of IRD in patients with musculoskeletal complaints. Our algorithm is expected to increase appropriateness of rheumatology referrals when aimed at use within primary care and sufficiently validated. To ensure successful implementation the barriers and facilitators for implementation need to be considered and identified from the perspective of patients and caregivers as in the present study. The majority of identified barriers and facilitators was overlapping between stakeholders. The current findings can reliably inform further decision strategies for implementation of the digital referral algorithm for inflammatory rheumatic diseases.

## Supplementary Information

**Additional file 1.**

## Data Availability

The data used and analysed during the current study are available from the corresponding author on reasonable request.

## Abbreviations

GP: General practitioner
IRD: Inflammatory rheumatic disease
MEC-U: Medical research Ethics Committees United
MSC: Musculoskeletal complaints
SD: Standard deviation
