# Supplementary material for: Barriers and facilitators for implementation of a digital referral algorithm for inflammatory arthritis - a qualitative assessment in patients and caregivers
Source: BMC Prim Care. 2022 Sep 26;23:248. doi: 10.1186/s12875-022-01858-w (PMC9510757; doi:10.1186/s12875-022-01858-w)
Supplement: Supplementary file 1 — Additional file 1. [file 12875_2022_1858_MOESM1_ESM.docx]

**Supplementary files**

1. **Question guide focus group patients**

**Opening question**

1. When have you first visited the rheumatologist and what was the outcome of that consultation?

**Introduction questions**

1. Which care trajectory did you follow to get to a consult with the rheumatologist?
2. Which care trajectory have you followed ever since the consult with the rheumatologist up until where you are now?

**Transition questions**

1. Which complaints did you experience in the beginning?
2. How long did it take you to visit the general practitioner because of your complaints?
3. Did you look for or receive any help before visiting the general practitioner, for example online information or information from a patient association?

**Key questions**

1. What is your opinion on providing an online referral tool for patients to use from home?
2. What added value could such a tool possibly have?
3. What would trigger you to make use of this tool?
4. What would trigger you to not make use of this tool?
5. What outcome would you like to get from using this tool?
6. How can we increase usability of the online referral tool?
7. What is needed for you to gain trust in this tool?
8. How do you think rheumatologists and general practitioners will react on this tool?
9. What do you think is needed for healthcare professionals to gain trust in this tool and its outcome and include it into their daily practice?
10. What other stakeholders should we include when designing an implementation strategy for this tool?
11. What are other possible barriers for implementation and use of this tool?

**Final questions**

1. Give a summary. Is this summary adequate or would you want to add something?
2. Have we missed anything during our discussion, which we can possibly address in next focus groups?
3. **Proposed digital referral algorithm to determine risk of IRD**


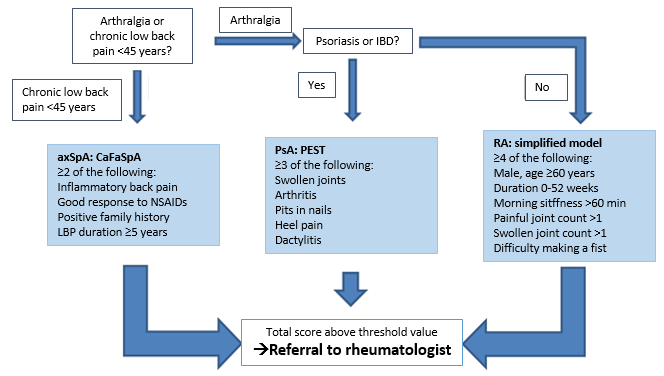


1. **Survey caregivers**

*Welcome to this online survey. We will first provide you with some background information concerning our research. Currently the focus of Dutch healthcare is shifting more towards providing the right care at the right place for each individual patient. However, it has proven to be difficult to recognise which patients are at risk of an inflammatory rheumatic disease and in need of visiting a secondary care rheumatologist. To overcome this difficulty, we have developed a digital referral algorithm which is able to provide patients with a first distinction on whether their musculoskeletal complaints appear to be of inflammatory nature. With this research we want to evaluate whether we can implement this algorithm for patients to use at home.*

1. What percentage of referred patients do you suspect of having an inflammatory rheumatic disease?

100%

0%

1. Do you think referrals of patients without an inflammatory complaint are a consequence of a lack of knowledge on inflammatory rheumatic conditions among patients?

🞏 Strongly agree

🞏 Agree

🞏 Neutral

🞏 Disagree

🞏 Strongly disagree

1. Do you think patients with musculoskeletal complaints look for information or advice on possible rheumatic complaints prior to visiting a general practitioner?

🞏 Strongly agree

🞏 Agree

🞏 Neutral

🞏 Disagree

🞏 Strongly disagree

1. To what extent do you think patients use the right information to decide whether to consult a healthcare professional?

100%

0%

*Patients experiencing musculoskeletal complaints are longing for a platform on which they can find accurate information and advice considering their complaints. Currently most often consulted platforms include the websites www.reumanederland.nl and [www.thuisarts.nl](http://www.thuisarts.nl).*

1. To what extent do you trust the currently available information for patients?

100%

0%

1. Do you consider it necessary to create more awareness on symptoms of inflammatory rheumatic conditions among patients?

🞏 Strongly agree

🞏 Agree

🞏 Neutral

🞏 Disagree

🞏 Strongly disagree

*We have developed a digital algorithm for use by patients with musculoskeletal complaints. This algorithm is believed to create more awareness on rheumatic diseases among patients and advise them on consulting a general practitioner. The digital algorithm is developed in a way that patients can fill it out themselves and use the outcome of the algorithm to propose a more directed question at their GP consultation. Consequently, the GP can, based on his or her own findings, together with the patient, decide whether or not to refer the patient towards a rheumatologist.*

1. What is your opinion on the digital referral algorithm?

|  | Strongly disagree | Disagree | Neutral | Agree | Strongly agree |
| --- | --- | --- | --- | --- | --- |
| The purpose of the referral tool is clear |  |  |  |  |  |
| The outcome of the referral tool includes sufficient information |  |  |  |  |  |
| The outcome of the referral tool would matter to me |  |  |  |  |  |
| The outcome of the referral tool is easily understood |  |  |  |  |  |
| I would trust the outcome of the referral tool |  |  |  |  |  |
| Patients can fill out this referral tool reliably |  |  |  |  |  |
| The referral tool would help me make decisions on further steps within the care cycle |  |  |  |  |  |
| It would take little effort to incorporate the outcome of the referral tool in a consult |  |  |  |  |  |

1. Which questions should definitely be included in the referral algorithm?

………………………………………………………………………………………………………………………………

1. Which information should the outcome of the referral algorithm definitely contain?

………………………………………………………………………………………………………………………………

1. What added value do you see for the use of this referral algorithm by patients?

………………………………………………………………………………………………………………………………

1. What barriers do you see for the use of this referral algorithm by patients?

………………………………………………………………………………………………………………………………

1. Do you have any other suggestions or remarks?

………………………………………………………………………………………………………………………………
